# Evidence of Horse Exposure to *Anaplasma phagocytophilum*, *Borrelia burgdorferi*, and *Leishmania infantum* in Greece through the Detection of IgG Antibodies in Serum and in an Alternative Diagnostic Sample—The Saliva

**DOI:** 10.3390/biom13091374

**Published:** 2023-09-11

**Authors:** Labrini V. Athanasiou, Eleni G. Katsogiannou, Panagiota Tyrnenopoulou, Dimitrios Gougoulis, Kosmas N. Apostolidis, Stavros M. Papadakis, Kassiopi Christina G. Kokkinaki, Vasileios G. Papatsiros, Constantina N. Tsokana

**Affiliations:** Veterinary Faculty, University of Thessaly, 43100 Karditsa, Greece; elkatsog@uth.gr (E.G.K.); ptyrnenop@uth.gr (P.T.); dgoug@vet.uth.gr (D.G.); kapostol@vet.uth.gr (K.N.A.); papasta68@gmail.com (S.M.P.); kaskokki@uth.gr (K.C.G.K.); vpapatsiros@vet.uth.gr (V.G.P.)

**Keywords:** *Anaplasma phagocytophilum*, *Borrelia burgdorferi*, horse, indirect fluorescence antibody test, *Leishmania infantum*, saliva

## Abstract

Among the various zoonotic pathogens that infect horses, *Anaplasma phagocytophilum, Borrelia* spp. and *Leishmania* spp. have gained scientific interest, and relevant molecular and serological studies in horses have been conducted worldwide. Moreover, human and veterinary medicine have extensively applied alternatives to serum diagnostic samples—such as saliva—for detecting pathogens or antibodies. In this study, we investigated the exposure of horses in Greece to *A. phagocytophilum*, *B. burgdorferi*, and *L. infantum*, and we assessed the diagnostic accuracy of saliva compared to serum in detecting IgG antibodies against the abovementioned pathogens. Paired saliva and serum samples were collected from 317 horses from different regions in Greece. The paired samples were examined using the indirect fluorescent antibody test (IFAT) for detecting IgG antibodies against *A. phagocytophilum, B. burgdorferi*, and *L. infantum.* Sensitivity, specificity, positive likelihood ratio (PLR), and negative likelihood ratio (NLR) were determined to assess the validity of saliva as an alternative to serum. The receiver operating characteristic (ROC) curve revealed that the optimal cut-off value for detecting antibodies against all the examined pathogens in saliva was 1/10. Higher seropositivity rates were found for *B. burgdorferi* (15.14%) and *A. phagocytophilum* (14.19%) compared to *L. infantum* (1.26%). The detection of IgG antibodies using IFAT in saliva samples had a good test performance compared to serum. The two sample types had a substantial to almost perfect agreement. Although the sensitivity was moderate (70.83–75.56%) in all cases, the specificity was almost perfect to perfect (99.63–100%). This study provides the first evidence that horses in Greece are exposed to *A. phagocytophilum* and *B. burgdorferi* and confirms that the seroprevalence of *L. infantum* in horses in Greece remains low. Our findings suggest that saliva sampling coupled with IFAT could be successfully applied for detecting IgG antibodies against these important zoonotic pathogens in large-scale epidemiological studies in horses, at the population level, as an alternative to serum.

## 1. Introduction

The close contact of horses with humans, the global increase in horse movements, and the subsequent potential to spread zoonotic and equine diseases, coupled with the economic importance of this species, resulted in the investigation of its role in the epidemiology of significant human and equine pathogens in a One Health context [1,2]. Among the various zoonotic pathogens that infect horses, *Anaplasma phagocytophilum*, *Borrelia* spp., and *Leishmania* spp. have gained scientific interest, and relevant reports on equine infection and/or exposure have been produced all over the world [3,4,5,6]. 

Equine granulocytic anaplasmosis caused by *A. phagocytophilum* leads to subclinical or clinical disease [7]. The latter manifests as acute febrile illness with loss of appetite, lethargy, hemorrhages, and lameness, which is usually self-limiting [8]. Thrombocytopenia is a common laboratory finding that results in characteristic petechial hemorrhages on the mucosal surface of the lips and gums [6]. Diagnosis is usually based on history, clinical signs, and laboratory findings [9]. *Anaplasma phagocytophilum* morulae appear in the cytoplasm of neutrophils during the acute phase of the disease and can be detected through microscopy [6]. Molecular methods are also helpful for the diagnosis of equine granulocytic anaplasmosis as well as the detection of subclinical infections. The indirect fluorescent antibody test (IFAT) and enzyme-linked immunosorbent assay (ELISA) are the serological methods most commonly employed for detecting anti-*A*. *phagocytophilum* antibodies in horses [6,8]. Although serology presents limitations in individual diagnostics of subclinical cases, seroprevalence studies provide essential information about the serological status of the horse population globally [6,8]. The recorded seroprevalences differ among the countries, ranging from 4% in Switzerland to 72.8% in the Czech Republic [8,10]. Notably, previous studies suggested that equine *A. phagocytophilum* strains are similar or identical to those causing disease in humans and dogs [11].

*Borrelia burgdorferi* infection is usually asymptomatic in horses but naturally occurring syndromes with a broad spectrum of clinical manifestations, including arthritis, lameness, anterior uveitis, encephalitis, and cutaneous pseudolymphoma, have been documented. Although the causal relationship has not been proven in most cases [8], and there is a paucity of conclusive clinical equine borreliosis data in Europe, several case reports of equine neuroborreliosis with *B. burgdorferi* detection in the central nervous system have been reported in North America [12,13]. Furthermore, dual infections with *A. phagocytophilum* [14,15,16] have also been recorded, and are considered to increase the disease severity [17]. Studies in various countries globally have reported serological evidence of exposure to *B. burgdorferi* s.l in horses: from 0% in different regions in Africa to 48% in France [18]. 

Since the first case of equine cutaneous leishmaniosis in 1927 in Argentina, several studies have reported equine subclinical and cutaneous leishmaniosis cases [4]; *Leishmania infantum* in the Old World and *L. (Viannia) braziliensis* in the New World were the most frequently involved species. *Leishmania infantum* was also reported in Brazil when Soares et al. identified three autochthonous cases of mixed infection with *L. braziliensis* [19]. *Leishmania (Mundinia) martiniquensis* infection was detected in horses in Germany [20] and Florida [21]. As for the seroprevalence studies in horses, their findings vary significantly among different countries depending on the geographical area and the serological method used; seroprevalence ranges from 0.3% for *L. infantum* in Greece to 76.3% for *L. (V.) braziliensis* in Paraná State, Brazil [22,23,24,25,26]. The clinical signs of the disease are similar irrespective of the geographical location and the responsible *Leishmania* species; single or multiple papules or nodules located in the eyes, muzzle, neck, pinnae, scrotum, and legs are the most frequently reported manifestations, while visceral lesions have not been described until now [4]. Horses are considered a resistant host to *Leishmania* infections since both subclinical and clinical diseases are characterized by a lack of antibodies or low antibody titers [4,27,28]. Among the different serological methods, IFAT, direct agglutination test (DAT), and ELISA were the most frequently used in seroprevalence studies in horses [4,22,23,24].

Oral fluid or saliva has been extensively used for detecting pathogens or antibodies in infected individuals; human immunodeficiency virus, measles, rubella, and hepatitis A, B, and C viruses are some of the applications in human medicine up to now [29,30,31]. The body of literature is also increasing in veterinary medicine with oral fluid or saliva samples being used for the detection of infectious agents such as foot-and-mouth disease virus in cattle and porcine reproductive and respiratory syndrome virus in pigs but also antibodies against *Actinobacillus pleuropneumoniae*, PRRSV, and *Toxoplasma gondii* in pigs [32,33,34], *L. infantum* in dogs [35], feline immunodeficiency virus (FIV) and *Bartonella henselae* [36,37] in cats, and *Gasterophilus intestinalis* and tapeworms in horses [38,39]. The different studies show variable results regarding the performance of this alternative sample type ranging from poor to perfect agreement with the traditional serum samples. 

The oral fluid or saliva sampling procedure is noninvasive, substantially easier, quicker, less stressful, and painless [39,40]. Although horses are generally tolerant to blood collection, oral fluid could be a useful alternative diagnostic sample type in cases where a massive collection of samples is needed in a straightforward and timely manner, such as epidemiological and surveillance studies and for the examination of show and racehorses, especially during international events. Moreover, oral fluid samples are easy to collect even from pet owners [39] or horse breeders, as adverse effects resulting from the sampling procedure and subsequent treatment recommendations from a qualified veterinarian are not anticipated. However, this alternative biological sample needs to be validated case-by-case against current gold standard methods to be used as a diagnostic tool. 

In this study, we provide for the first time seropositivity data against *A. phagocytophilum* and *B. burgdorferi* in horses in Greece, and we report updated data on the seroprevalence of *L. infantum* in horses from different regions of the country. We also assess the diagnostic accuracy of saliva compared to serum when using IFAT to detect antibodies against these three important zoonotic pathogens in horses. 

## 2. Materials and Methods

### 2.1. Animals

A total of 317 domestic horses aged 1 to 15 years old from two regions of Greece, Thessaly and Crete, were enrolled in the study. The horses were categorized into age ranges as follows: young (≤3 years), adult (>3 and <12 years), or senior (≥12 years). The regions are located at about 39°40′ Ν, 22°30′ Ε and 35°20′ N, 24°30′ E, respectively. The climate in Thessaly is continental, with a cool winter and warm summer, while in Crete, it is typically Mediterranean, with a mild winter and warm and dry summer. The horses were clinically healthy at the sampling time, except for being infested by ticks. The sampling took place from spring to summer. 

### 2.2. Serum and Saliva Sampling 

Saliva samples were collected using a Salivette^®^ (Sarstedt, Nümbrecht, Germany) saliva collection kit as previously described [40], with minor modifications. In brief, following washing with tap water to remove any feed remnants, the cotton swab was maintained in the horse’s mouth vestibule for 30–40 s over and under the tongue, across the third or fourth maxillary premolar. Paired blood samples were obtained with an 18-G needle and 1 1/2 inches from the external jugular vein into a vacutainer tube (BD, Franklin Lakes, NJ, USA), without anticoagulant, for serum collection.

The blood and saliva samples collected in the nearby areas were promptly transferred directly to the Laboratory of the Clinic of Medicine, Faculty of Veterinary Medicine, School of Health Sciences, University of Thessaly, Greece. The samples collected from distant regions were centrifuged in local veterinary clinics, and then transported to the Laboratory of the Clinic of Medicine for further analysis and processing. 

The blood samples were subjected to centrifugation at 400× *g* for 10 min, allowing for the separation of serum. The obtained serum samples were then transferred to plastic vials and refrigerated at −20 °C pending analysis. The saliva samples underwent centrifugation at 3000× *g* for 10 min at 4 °C and were subsequently stored at −80 °C until further testing.

### 2.3. IFAT in Serum and Saliva Samples

The serum and saliva samples were tested for the presence of IgG antibodies against *A. phagocytophilum*, *B. burgdorferi*, and *L. infantum* using IFAT. The IFAT was performed following the instructions provided by the manufacturer (MegaFLUO^®^ ANAPLASMA ph., MegaFLUO^®^ BORRELIA horse, and MegaFLUO^®^ LEISH, Horbranz, Austria). The positive and negative controls with the respective conjugates provided by the manufacturer were used in each analysis. In the case of serum samples, cut-off values of 1/80 and 1/64 were used for *A. phagocytophilum* and *B. burgdorferi*, respectively, based on the instructions provided. For *L. infantum*, a cut-off value of 1/40 was employed as previously described [24]. As for the saliva samples, three dilutions were tested for all three pathogens: 1/10, 1/20, and 1/40. The observations were made using a Nikon Eclipse E-400 fluorescence (Nikon, Badhoevedorp, The Netherlands) microscope with a 100× objective. 

### 2.4. Statistical Analysis 

Sensitivity, specificity, positive likelihood ratio (PLR), and negative likelihood ratio (NLR) were determined to assess the validity of detecting antibodies against *A. phagocytophilum*, *B. burgdorferi*, and *L. infantum* in saliva compared to serum, using the MedCalc Statistical Software version 14.8.1 (MedCalc Software bvba, Ostend, Belgium)). PLR values > 10 and NLR values < 0.1 indicated good performance [41]. The agreement between the antibody detection results in the two biological materials was measured using Cohen’s kappa (κ) value [42,43]. A value of 0 to 1 indicated a fair to perfect agreement, respectively. The same software was used to generate receiver operating characteristic (ROC) curves and areas under the curve (AUCs) and to determine the optimal threshold for the cut-off values used to detect antibodies against the previously mentioned pathogens. The accuracy based on the AUC values was categorized as low (0.5 < AUC ≤ 0.7), moderate (0.7 < AUC ≤ 0.9), or high (0.9 < AUC ≤ 1) [44]. The “N−1” chi-squared test was employed to determine the significance of the difference in the percentage of seropositivity among different age groups [45,46], using the abovementioned statistical software.

## 3. Results

Table 1 shows the numbers and percentages of positive samples for IgG antibodies against *A. phagocytophilum*, *B. burgdorferi*, and *L. infantum* and their combinations in both serum and saliva samples. Compared to serum, the number of positive saliva samples for anti-*A. phagocytophilum* and anti-*B. burgdorferi* antibodies was lower for all three cut-off values. Similarly, the number of positive saliva samples for anti-*L. infantum* antibodies was lower for the 1/20 and 1/40 cut-off values. However, the number of positive saliva samples for anti-*L. infantum* antibodies was the same as in serum for the 1/10 cut-off value. In all cases, the use of higher cut-off values resulted in a lower number of positive samples. Co-exposure was observed in six horses and concerned only *A. phagocytophilum* and *B. burgdorferi*.

Table 2 summarizes the number of positive serum samples per age group for IgG antibodies against *A. phagocytophilum*, *B. burgdorferi*, *L. infantum*, and their combinations. Statistical analysis showed that senior horses presented statistically significantly higher seropositivity for *A. phagocytophilum* and *B. burgdorferi* than the other two age categories. Similarly, the seropositivity rates for the co-exposure to *A. phagocytophilum* and *B. burgdorferi* were statistically significantly different among the three age groups with the senior group presenting the highest number of seropositive horses for both pathogens. 

The sensitivity, specificity, PLR, and NLR values for detecting anti-*A. phagocytophilum*, anti-*B. burgdorferi*, and anti-*L. infantum* antibodies in saliva when using three different cut-off values and when considering the detection of antibodies in serum as the reference method are presented in Table 3a–c. 

When using a cut-off value of 1/10, the sensitivity for detecting antibodies against *A. phagocytophilum* and *L. infantum* (75.56% and 75.00%, respectively) was higher than that for *B. burgdorferi* (70.83%). As for the specificity, it was perfect for detecting anti-*B. burgdorferi* antibodies, and almost perfect for detecting antibodies against the other two pathogens (99.63% and 99.98%, respectively). When a cut-off value of 1/20 was used, the sensitivity for detecting antibodies against *A. phagocytophilum* and *B. burgdorferi* was similar (64.44% and 64.58%, respectively), while it was 50% for detecting anti-*L. infantum* antibodies. The sensitivity for all the pathogens was even lower for the cut-off value of 1/40. On the contrary, the specificity was perfect or almost perfect when the 1/20 and 1/40 cut-off values were used. 

The PLR values for detecting antibodies against *A. phagocytophilum* (cut-off values of 1/10 and 1/20) and *L. infantum* (cut-off value of 1/10) were above 10. PLR value could not be calculated in in all the other cases that specificity was 100%, because specificity is the denominator (1-specificity) of the equation. In all these cases, the test performance was good based on PLR values, while the best NLR value was observed for the detection of anti-*A. phagocytophilum* and anti-*L. infantum* antibodies (0.25) when the cut-off value was 1/10.

As shown in Table 4, there was an almost perfect agreement between serum and saliva samples for detecting antibodies against *A. phagocytophilum* and *B. burgdorferi* (>0.80) with a cut-off value of 1/10. In all the other cases, there was a substantial agreement (0.61–0.80), except for detecting anti-*L. infantum* antibodies with a 1/40 cut-off value in which the agreement was fair (0.21–0.40).

The number of true and false positive and negative samples for detecting antibodies against the three pathogens in serum and saliva, with the results in serum taken as a reference, is presented in Table 5. Using lower cut-off values resulted in an increase in true positive samples for detecting anti-*A. phagocytophilum* and anti-*B. burgdorferi* antibodies and a decrease in false negatives. False positive results were recorded for the anti-*A. phagocytophilum* and anti-*L. infantum* antibodies, with cut-off values of 1/10 and 1/20, and 1/10, respectively.

**Figure 1:**
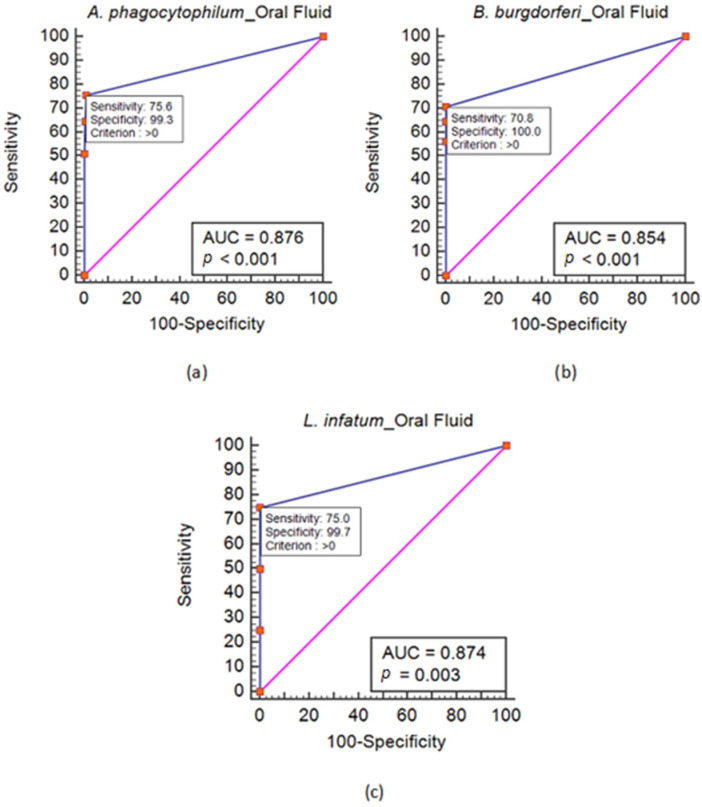
Receiver operating characteristic (ROC) curve analysis for detecting antibodies against *A. phagocytophilum* (**a**), *B. burgdorferi* (**b**), and *L. infantum* (**c**) in saliva.

Figure 1 shows the ROC curve analysis for detecting antibodies against these three pathogens in saliva. The ROC analysis revealed that the cut-off value closest to the upper-left corner of the AUC plot corresponded to the optimal criterion (>0). The sensitivity and specificity of these spots indicate that the best cut-off value was 1/10. Moreover, the accuracy interpreted by the AUC values was categorized as moderate (0.7 < AUC ≤ 0.9). 

## 4. Discussion

In this study, we showed that IgG antibodies against *A. phagocytophilum*, *B. burgdorferi*, and *L. infantum* circulate in detectable levels in equine saliva samples. We also determined the diagnostic accuracy of this alternative sample type compared to serum, using IFAT. Based on the serological investigation, horses in Greece are exposed to *A. phagocytophilum*, *B. burgdorferi*, and *L. infantum*. Co-exposure was observed in six horses, and it only concerned *A. phagocytophilum* and *B. burgdorferi*. Senior horses (≥12 years of age) presented higher seropositivity rates against *A. phagocytophilum* and *B. burgdorferi* separately as well as in combination compared to the other two age groups. We herein provide, for the first time, seropositivity data against *A. phagocytophilum* and *B. burgdorferi* in horses in Greece, and we also report updated data on the seroprevalence of *L. infantum* in this animal species from different regions of the country. 

The ROC analysis revealed that the 1/10 cut-off value was optimal for detecting antibodies against all the examined pathogens, taking the results in serum as a reference. Moreover, we chose to evaluate the performance of the saliva samples in detecting antibodies by measuring the PLR and NLR values. These values are independent of the disease prevalence, and they do not vary among the different populations and settings [41]. According to the PLR values for the 1/10 cut-off value, the detection of anti-*A. phagocytophilum*, anti-*B. burgdorferi*, and anti-*L. infantum* antibodies using IFAT in saliva samples had a good test performance compared to serum. Antibodies against the abovementioned pathogens will likely be detected in saliva samples in seropositive animals. The best (lowest) NLR values were observed for detecting anti-*A. phagocytophilum* and anti-*L. infantum* antibodies. However, the NLR values were, in all cases, indicative of a moderate power to identify seronegative animals when saliva samples were negative. Thus, a negative saliva sample cannot rule out seropositivity. Overall, the two sample types had an almost perfect agreement in detecting antibodies against *A. phagocytophilum* and *B. burgdorferi*, while there was a substantial agreement in detecting anti-*L. infantum* antibodies. The sensitivity values were higher for detecting antibodies against *A. phagocytophilum* and *L. infantum* than anti-*B. burgdorferi* antibodies. Although the sensitivity was moderate in all cases, the specificity was perfect for detecting anti-*B. burgdorferi* antibodies, and almost perfect for detecting antibodies against the other two pathogens. 

In cats, saliva samples were less sensitive than serum for detecting anti-*B. henselae* antibodies when using IFAT. In that case, saliva was considered of limited utility for epidemiological or diagnostic purposes since oral fluid antibodies were detected more often in cats with high *B. henselae* serum antibody titers [36]. The use of saliva for the detection of anti-*Mycobacterium bovis* IgG antibodies by dual path platform (DPP) in experimentally infected cattle also had a low diagnostic value due to relatively low specific IgG levels (100–300 times lower than paired serum samples) and variable detection rates [47]. Additionally, anti-*Salmonella* antibodies were not detected in oral fluid samples from pigs, while the paired serum samples were positive. In the same study, the detection of specific antibodies against the hepatitis E virus showed poor agreement with serum samples at the first sampling and significant agreement in the second sampling [48].

On the contrary, a modified protocol of a commercial ELISA for detecting anti-*Salmonella* IgG antibodies in pig saliva samples showed a moderate correlation with the application of the assay in paired serum samples. Although antibody levels in pig sera were consistently higher, anti-*Salmonella* IgG antibodies were detected in all the matching saliva samples. Thus, the authors suggested that individual saliva samples can represent a suitable alternative to blood samples for detecting anti-*Salmonella* antibodies at an individual pig level [49]. Seemingly, when saliva samples were used to detect IgG antibodies against the FIV using IFAT and Western blot, they successfully identified the seropositive cats. However, it is worth mentioning that higher ratios of salivary IgG compared to serum IgG were found in both FIV seronegative and seropositive cats with oral lesions compared to cats without oral lesions, suggesting a possible link between positive oral fluid samples and the presence of oral inflammatory lesions [50]. 

Similarly, in a mixed population of vaccinated and unvaccinated cats against FIV, commercial rapid tests, based on immunochromatography, accurately diagnosed FIV infection using saliva, irrespective of FIV vaccination history [37]. The detection of antibodies against *L. infantum* in canine oral transudate using ELISA showed almost perfect to moderate agreement with serum samples. The authors suggested that this sample type could be used for sick dogs with high antibody titers, but the results are expected to be less optimal in apparently healthy dogs [35]. Likewise, IgG2 antibodies against *L. infantum* were highly correlated between saliva and serum samples using time-resolved immunofluorometric assays, and the reduction in antibody levels was related to clinical improvement [51]. 

In another study in horses, Lightbody et al. evaluated the performance of a commercially available test for tapeworm infections based on detecting tapeworm-specific IgG antibodies in saliva. The test showed similar sensitivity to the applied modified fecal egg count analysis when the parasitic load was high (>20 tapeworms). Notably, the assay sensitivity was higher for low parasitic burdens (>one tapeworm), possibly due to the inability of the fecal egg count method to identify immature tapeworms [38]. Likewise, in an earlier study, the saliva test was as accurate as ELISA for diagnosing low parasitic load (>one tapeworm) [52]. The authors suggested that this easy-to-use test could be widely deployed for regular tapeworm testing to reduce the frequency of praziquantel and pyrantel pamoate administration in horses [38,52]. 

The presence of the different antibody classes in oral fluid is not surprising. Oral fluid comprises saliva and serum transudates from capillaries in the oral mucosa and gingival tissues. Blood components (e.g., immunoglobulins) access the mucosal surface through the junction between the teeth and the mucosa. Immunoglobulins in oral fluid originate from the passage of the systemic immune system’s serum antibodies (mainly IgG, but also IgA and IgM) [53,54,55]. However, they are also produced locally (mainly IgA but also IgG and IgM at low levels). Local production is based on the blood-derived plasma cells of the secretory immune system in the major salivary glands and duct-associated lymphoid tissue of minor salivary glands distributed around the oral cavity [32,55]. 

This study examined the oral fluid samples only for IgG antibodies against *A. phagocytophilum*, *B. burgdorferi*, and *L. infantum*. However, previous animal studies have successfully detected IgA and IgM antibodies and showed the diagnostic utility of the different antibody classes. In horses, the researchers in one study evaluated the performance of a self-developed ELISA for detecting specific anti-*G. intestinalis* L3 IgG, IgM, and IgA antibodies using paired serum and saliva samples [39]. The IgA class—the main immunoglobulin class in mucosal secretions—showed the highest reaction intensity. The salivary IgG and the IgA-specific antibody reactivity was significantly higher in moderate and severe infection (>50 larvae) than in healthy horses. On the contrary, a low parasitic burden (10–50 larvae) status could not be differentiated from an infection-free status based on the IgA intensity of reaction. As for the IgM antibodies, the same level of reactivity was seen only in severely infected horses (>200 larvae). Therefore, the authors suggested that IgG and IgA antibody activity in saliva may be used for detecting horses moderately and severely infested with *G. intestinalis* [39]. 

Another study demonstrated that the mucosal IgA antibody response arises earlier than the systemic response and suggested that measuring mucosal antibodies in saliva could be applied to identify pigs infected with *A. pleuropneumoniae* at an early stage of infection [34]. Regarding the detection of *T. gondii* antibodies in oral fluid pig samples, the IgA class performed better than the IgG class. The authors suggested that this sample type, coupled with the ELISA test, could be successfully applied as a screening method at the farm level. However, its use for determining the serological status in individual animals was not supported by the findings of that study [32]. In other studies in pigs, IgG in oral fluid samples presented better diagnostic performance compared to IgA antibodies for porcine reproductive and respiratory syndrome virus [33], porcine circovirus type 2 [56], classic swine fever virus [57], and *Lawsonia intracellularis* infections [58]. Similarly, cats with chronic gingivostomatitis had significantly higher salivary IgM and IgG but significantly lower salivary IgA concentrations than healthy cats [59].

Many factors may influence the accuracy of oral fluid or saliva as a diagnostic tool: the targeted pathogen, the stage of infection, the method deployed, the sample collection device, and the age-related decrease in specific antibody levels [29,31,36,39,47,49,60]. In a recent study, the researchers showed that the presence of feed in horse saliva could affect the measurement of selected biomarkers. The type of the collection device (cotton or sponge) could also influence the results obtained. They suggested that clean saliva and the same collection device should ensure consistent measurements [61]. As for the IgG antibody component in oral fluid samples, it has been suggested that dental status did not affect the sensitivity and specificity values of an assay used for detecting IgG antibodies against rubella and hepatitis A viruses in humans [62]. 

Moreover, other studies have shown that storing oral fluid samples at environmental temperatures does not affect their integrity. When human saliva samples—collected with a specific device—were stored for one month in ambient temperatures in a tropical country, the antibodies against HIV were successfully preserved [30]. Similarly, anti-PRRSV antibodies in pig oral fluid were relatively resistant to degradation over 12 days, and the authors suggested that appropriate specimen-handling protocols, including prompt freezing or refrigeration at 4 °C, would maintain the integrity of anti-PRRSV antibodies in oral fluid samples [63]. 

In this study, we report serological data for *A. phagocytophilum*, *B. burgdorferi*, and *L. infantum* in horses from different regions in Greece. With regard to *A. phagocytophilum*, the recorded seroprevalences differ among the countries, ranging from 4% in Switzerland to 72.8% in the Czech Republic [8,10]. Variation in seropositivity rates is apparent in studies conducted in the same country: 11.3–18.0% in France [18,64] and 8.0–16.9% in Italy [65,66]. The seropositivity reported in our study (14.2%) is close to the data reported in other European countries. 

Regarding *B. burgdorferi*, this is the first study to report the exposure of horses in Greece, and the recorded rate was 15.4%. Studies in various countries globally have reported serological evidence of exposure to *B. burgdorferi* s.l. in horses, ranging from 0% in different regions in Africa [18] to 15.5% in Bulgaria [16], 16.8% in Sweden [67], 18.8% in Romania [14], and 16.1% in Germany [68], and even higher rates of 29% in Denmark [69], 33% in southwest Virginia [70], 45.1% in the northeastern United States [71], 47.8% in Slovakia [72], and 48% in France [18]. Limited data exist on the occurrence of *B. burgdorferi* in Greece and no human cases have been recorded. The studies conducted up to now showed a low seroprevalence of 3.3% in humans [73] and 0.1–2.2% in dogs [74,75]. Recently, a study showed that sheep are also exposed to *B. burgdorferi* in Greece and provided evidence of co-exposure with *A. phagocytophilum* [76].

The co-exposure observed in 1.89% of the examined horses was exclusively for *A. phagocytophilum* and *B. burgdorferi*, which also share a common vector. While this finding could potentially be attributed to cross-reaction between the two agents in IFAT, the low number of horses that reacted to both agents weakens this hypothesis. 

Senior horses presented higher seropositivity rates compared to adult and young horses for *A. phagocytophilum* and *B. burgdorferi* as well as for co-exposure to both agents. This finding is not surprising since older horses would have been exposed to vectors and their pathogens for prolonged periods of time compared to young and adult horses. 

A lower seropositivity rate was previously reported in the country for *L. infantum* (0.3%) [22] compared to our findings (1.26%). Similar low seroprevalences have been recorded in other countries, including Israel (1.4%) [23], Portugal (4%), [77] and Italy (6.5%) [78]. However, a recent study in Italy reported a seroprevalence of 13.9% [24], suggesting the heterogeneity of serological findings even in the same country. Moreover, a seroprevalence of 27% was recorded in Brazil for *L. infantum* [25] and as high as 76.3% in Paraná State, Brazil, for *L. (V.) braziliensis* [26]. 

Vector-borne diseases in horses can exact a heavy economic toll, causing substantial losses in veterinary expenses, treatment costs, and reduced performance. Additionally, exposure to these pathogens can impact local economies by affecting tourism and horse-related outdoor activities [79]. Hence, disease prevention measures to collectively minimize the risk of exposure (i.e., the use of effective repellents, grooming practices, and alterations in pasture management) in urban and peri-urban environments could yield substantial long-term savings and prevent potential losses, safeguarding the equine industry’s financial stability. The horses included in this study inhabited regions where environmental factors encourage the proliferation of ticks and sandflies.

Concerning the vector distribution and abundance in Greece, several studies have shown that distinct populations of ticks and sandflies exist throughout the country. A total of 26 hard tick species and subspecies have been identified in animals and humans in Greece including *Ixodes ricinus* and *Rhipicephalus sanguineus* infected with *A. phagocytophilum* and *A. platys* [80,81,82]. Based on the existing country-wide studies, *I. ricinus*, the common vector of *B. burgdorferi* and *A. phagocytophilum*, is not among the most prevalent tick species in the country, representing 0.07% of the ticks collected from sheep in mainland Greece and the Aegean islands [83], 2.25% from sheep, 10.47% from cattle, 3.65% from goats, and 8.41% from dogs [84] and the 15.63% from goats in northern Greece [81]. However, in two studies conducted in northern Greece, *I. ricinus* represented 46% [85] and 50.50% [86] of the ticks collected from sheep and goats, respectively. 

Similarly, the entomological studies conducted in Greece identified eleven species belonging to the genus *Phlebotomus* spp. Among them, the proven or suspected vectors for *L. infantum—P. perfiliewi*, *P. tobbi*, and *P. neglectus*—have been repeatedly recorded [87,88,89]. The previous studies reported an infection prevalence of *L. infantum* in sandflies ranging from 0.41% [88] to 5.4% [90] in Attiki, Central Greece, and as low as 0.12% on the island of Corfu [91]. However, a recent study in two refugee camps in the region of Thessaloniki, in northern Greece, showed an unusually high prevalence of infection in *P. perfiliewi*, *P. tobbi*, and *P. simici* ranging from 43% to 52% [92]. 

Although data gathering on vectors is not systematic in Greece, the undertaken studies show that ticks can transmit *A. phagocytophilum* and *B. burgdorferi*, and sandfly species that are competent vectors of *L. infantum* are widely distributed throughout the country [80,88]. Conducting further studies on the mainland and islands of Greece would be valuable in determining the level of exposure of different animal species and humans to these pathogens, as well as the rate of infection among vectors. 

## 5. Conclusions

Our study showed that horses in Greece are exposed to *A. phagocytophilum*, *B. burgdorferi*, and *L. infantum.* The IgG antibodies against *A. phagocytophilum*, *B. burgdorferi*, and *L. infantum* circulate in equine saliva at detectable levels. Our findings suggest that when antibodies against the abovementioned pathogens are detected in saliva samples it is nearly certain that the animal is also seropositive. Thus, saliva sampling coupled with IFAT could be successfully applied in large-scale serological studies at the population level as an alternative to serum.

## Figures and Tables

**Table 1:** The numbers and the percentages of positive samples for IgG antibodies against *A. phagocytophilum*, *B. burgdorferi*, and *L. infantum* and their combinations in both serum and saliva samples at the corresponding cut-off values.

Pathogen	Serum	Saliva		
		Cut-Off		
		1/10	1/20	1/40
*A. phagocytophilum*	45/317 (14.20%) ^a^	35/317 (11.04%) ^a,b^	30/317 (9.46%) ^a,b^	23/317 (7.23%) ^b^
*B. burgdorferi*	48/317 (15.14%) ^a^	34/317 (10.73%) ^a.b^	31/317 (9.78%) ^b^	27/317 (8.25%) ^b^
*L. infantum*	4/317 (1.26%) ^a^	4/317 (1.26%) ^a^	2/317 (0.63%) ^a^	1/317 (0.32%) ^a^
*A. phagocytophilum +* *B. burgdorferi*	6/317 (1.89%) ^a^	4/317 (1.26%) ^a^	2/317 (0.63%) ^a^	1/317 (0.32%) ^a^
*A. phagocytophilum +* *L. infantum*	0%	0%	0%	0%
*B. burgdorferi +* *L. infantum*	0%	0%	0%	0%
*A. phagocytophilum +* *B. burgdorferi +* *L. infantum*	0%	0%	0%	0%

^a,b^ Different superscripts next to percentages in different columns of the same row denote significant difference, *p* < 0.05.

**Table 2:** The numbers and percentages of positive samples per age group for IgG antibodies against *A. phagocytophilum*, *B. burgdorferi*, and *L. infantum* and their combinations in serum.

	Age Group Seropositive N * (%)		
Pathogen	≤3 Years Old	3–12 Years Old	≥12 Years Old
*A. phagocytophilum*	3 (6.7%) ^a^	25 (12.0%) ^a^	17 (26.6%) ^b^
*B. burgdorferi*	2 (4.4%) ^a^	26 (12.5%) ^a^	20 (31.3%) ^b^
*L. infantum*	0 (0%) ^a^	2 (1.0%) ^a^	2 (3.1%) ^a^
*A. phagocytophilum + B. burgdorferi*	0 (0%) ^a,b^	2 (1.0%) ^a^	4 (6.3%) ^b^
*A. phagocytophilum +* *L. infantum*	0 (0%)	0 (0%)	0 (0%)
*B. burgdorferi +* *L. infantum*	0 (0%)	0 (0%)	0 (0%)
*A. phagocytophilum +* *B. burgdorferi +* *L. infantum*	0 (0%)	0 (0%)	0 (0%)

* N = Number. ^a,b^ Different superscripts next to percentages in different columns of the same row denote significant difference, *p* < 0.05.

**Table 3:** (**a**) Sensitivity, specificity, positive likelihood ratio (PLR), and negative likelihood ratio (NLR) for detecting antibodies against *A. phagocytophilum*, *B. burgdorferi*, and *L. infantum* in saliva with a cut-off value of 1/10, taking the results in serum as a reference. (**b**) Sensitivity, specificity, positive likelihood ratio (PLR), and negative likelihood ratio (NLR) for detecting antibodies against *A. phagocytophilum*, *B. burgdorferi*, and *L. infantum* in saliva with a cut-off value of 1/20, taking the results in serum as a reference. (**c**) Sensitivity, specificity, positive likelihood ratio (PLR), and negative likelihood ratio (NLR) for detecting antibodies against *A. phagocytophilum*, *B. burgdorferi*, and *L. infantum* in saliva with a cut-off value of 1/40, taking the results in serum as a reference.

**(a)**			
	***A. phagocytophilum***	***B. burgdorferi***	***L. infantum***
Sens. % (95% CI)	75.56 (60.46–87.12)	70.83 (55.94–83.05)	75.00 (19.41–99.37)
Spec. % (95% CI)	99.63 (97.97–99.99)	100.00 (98.64–100.00)	99.68 (98.23–99.99)
PLR (95% CI)	205.51 (28.85–1464.02)	-	234.75 (30.62–1800.00)
NLR (95% CI)	0.25 (0.15–0.41)	0.29 (0.19–0.45)	0.25 (0.05–1.37)
**(b)**			
	***A. phagocytophilum***	***B. burgdorferi***	***L. infantum***
Sens. % (95% CI)	64.44% (48.78–78.13)	64.58% (49.46–77.84)	50.00 (6.76–93.24)
Spec. % (95% CI)	99.63 (97.97–99.99)	100.00 (98.64–100.00)	100.00 (98.83–100.00)
PLR (95% CI)	175.29 (24.48–1254.92)	-	-
NLR (95% CI)	0.36 (0.24–0.53)	0.35 (0.24–0.52)	0.50 (0.19–1.33)
**(c)**			
	***A. phagocytophilum***	***B. burgdorferi***	***L. infantum***
Sens. % (95% CI)	51.11% (35.77–66.30)	56.25% (41.18–70.52)	25.00 (0.63–80.59)
Spec. % (95% CI)	100.00 (98.65–100.00)	100.00 (98.64–100.00)	100.00 (98.83–100.00)
PLR (95% CI)	-	-	-
NLR (95% CI)	0.49 (0.36–0.66)	0.44 (0.32–0.60)	0.75 (0.43–1.32)

**Table 4:** Agreement between the detection of antibodies against *A. phagocytophilum*, *B. burgdorferi*, and *L. infantum*, in serum and saliva samples, taking the results in serum as a reference.

	Cut-Off	κ Value (95% CI)		
Pathogen		1/10	1/20	1/40
*A. phagocytophilum*		0.829 (0.735–0.923)	0.744 (0.630–0.859)	0.642 (0.507–0.777)
*B. burgdorferi*		0.805 (0.707–0.903)	0.756 (0.646–0.865)	0.686 (0.562–0.809)
*L. infantum*		0.747 (0.408–1.000)	0.664 (0.226–1.000)	0.397 (−0.145–0.939)

**Table 5:** Number of true and false positive and negative samples for detecting antibodies against *A. phagocytophilum*, *B. burgdorferi*, and *L. infantum* in saliva samples, using different cut-off values and taking the results in serum as a reference.

	*A. phagocytophilum*			*B. burgdorferi*			*L. infantum*		
Cut-Off Value	1/10	1/20	1/40	1/10	1/20	1/40	1/10	1/20	1/40
TP	34	29	23	34	31	27	3	2	1
FP	1	1	0	0	0	0	1	0	0
TN	271	271	272	269	269	269	312	313	313
FN	11	16	22	14	17	21	1	2	3

TP: true positive; FP: false positive; TN: true negative; FN: false negative.

## Data Availability

The data presented in this study are available on request from the corresponding author. The data are not publicly available due to the further processing required by other studies.
